# β-Hydroxybutyrate suppresses inflammasome formation by ameliorating endoplasmic reticulum stress *via* AMPK activation

**DOI:** 10.18632/oncotarget.12119

**Published:** 2016-09-19

**Authors:** Ha Ram Bae, Dae Hyun Kim, Min Hi Park, Bonggi Lee, Min Jo Kim, Eun Kyeong Lee, Ki Wung Chung, Seong Min Kim, Dong Soon Im, Hae Young Chung

**Affiliations:** ^1^ Molecular Inflammation Research Center for Aging Intervention, College of Pharmacy, Pusan National University, Geumjeong-gu, Busan, Republic of Korea

**Keywords:** β-hydroxybutyrate, endoplasmic reticulum, aging, inflammasome, AMPK, Gerotarget

## Abstract

β-Hydroxybutyrate, a ketone body that is used as an energy source in organs such as the brain, muscle, and heart when blood glucose is low, is produced by fatty acid oxidation in the liver under the fasting state. Endoplasmic reticulum (ER) stress is linked with the generation of intracellular reactive oxygen species and the accumulation of misfolded protein in the ER. ER stress is known to induce the NOD-like receptor protein 3 inflammasome, which mediates activation of the proinflammatory cytokine interleukin-1β, whose maturation is caspase-1-dependent. We investigated whether β-hydroxybutyrate modulates ER stress, inflammasome formation, and insulin signaling. Sprague Dawley rats (6 and 24 months of age) that were starved for 3 d and rats treated with β-hydroxybutyrate (200 mg·kg^−1^·d^−1^ i.p., for 5 d) were used for *in vivo* investigations, whereas human hepatoma HepG2 cells were used for *in vitro* studies. Overexpression of AMPK in cultured cells was performed to elucidate the molecular mechanism. The starvation resulted in increased serum β-hydroxybutyrate levels with decreased ER stress (PERK, IRE1, and ATF6α) and inflammasome (ASC, caspase-1, and NLRP3) formation compared with non-fasted 24-month-old rats. In addition, β-hydroxybutyrate suppressed the increase of ER stress- and inflammasome-related marker proteins. Furthermore, β-hydroxybutyrate treatment increased the expression of manganese superoxide dismutase and catalase *via* the AMP-activated protein kinase-forkhead box protein O3α transcription factor pathway both *in vivo* and *in vitro*. The significance of the current study was the discovery of the potential therapeutic role of β-hydroxybutyrate in suppressing ER-stress-induced inflammasome formation.

## INTRODUCTION

Ketone bodies are small lipid-derived molecules that serve as a circulating energy source for tissues in times of fasting or prolonged exercise [1]. The ketone bodies refer to three molecules: acetoacetate, β-hydroxybutyrate, and acetone. The majority of the ketone bodies are produced in the liver, although smaller amounts may be produced in other tissues [2]. In the liver, the acetyl-CoA generated from fatty acid oxidation is diverted away from the tricarboxylic acid cycle and converted into β-hydroxybutyrate through ketogenesis in the mitochondria [3].

β-Hydroxybutyrate production is regulated by the enzyme 3-hydroxy-3-methylglutaryl-CoA synthase (HMGCS2) in the mitochondria [4]. Most of the acetoacetate molecules are metabolized by β-hydroxybutyrate dehydrogenase to β-hydroxybutyrate [5]. Many studies have shown that ketosis causes accelerated aging. Thus, the increased ketone levels in the body cause age-related metabolic diseases [1]. However, some studies have shown that ketone bodies in low concentration have an anti-inflammatory effect. β-Hydroxybutyrate increases both manganese superoxide dismutase (MnSOD) and forkhead O bax transcription factor3 (FOXO3) which are antioxidant genes [6]. Ketone bodies also mediate the neuroprotective effects of calorie restriction (CR) [7]. In humans, the basal serum level of β-hydroxybutyrate is in the low micromolar range, but begins to rise to 1-2 mM after 2 d of fasting [2] and 6-8 mM with prolonged starvation [8].

The endoplasmic reticulum (ER) is a multifunctional organelle that co-ordinates protein folding, lipid biosynthesis, and calcium storage and release. Perturbations that disrupt ER homeostasis lead to the misfolding of proteins, ER stress, and upregulation of the ER stress response pathway, which is also known as the unfolded protein response (UPR) [9]. ER stress could be activated in the aging process through increased oxidative stress, the accumulation of harmful protein modifications, the misfolding and aggregation of proteins, and impairments in protein synthesis [10]. In addition, the protein cleansing system becomes impaired during aging as a result of the reduction in autophagic and proteasomal degradation [11]. In a recent study, ER stress inducers, including tunicamycin and thapsigargin, activated the NOD-like receptor protein 3 (NLRP3) inflammasome in human and murine macrophages [12]. The NLRP3 inflammasome is a molecular platform that activates proinflammatory cytokines such as interleukin-1β (IL-1β).

AMP-activated protein kinase (AMPK) is a sensor of the cellular energy level. AMPK increases the transcription activity of forkhead box protein O (FOXO) transcription factors, perhaps *via* direct phosphorylation, leading to resistance to oxidative stress and the extension of longevity [13]. FOXO3a can reduce the level of cellular oxidative stress by directly increasing the mRNA and protein levels of MnSOD and catalase [14].

The purpose of this study was to investigate the anti-inflammatory effects of the β-hydroxybutyrate in aged rats and cultured cells. This study evaluated the mechanism of the β-hydroxybutyrate mediated modulation of inflammasome formation through the amelioration of ER stress *via* AMPK activation. The significance of the current study was the finding of the potential therapeutic role of β-hydroxybutyrate in suppressing ER-stress-induced inflammasome formation.

## RESULTS

### Acute starvation inhibitd the inflammasome by ameliorating ER stress

**Figure 1:**
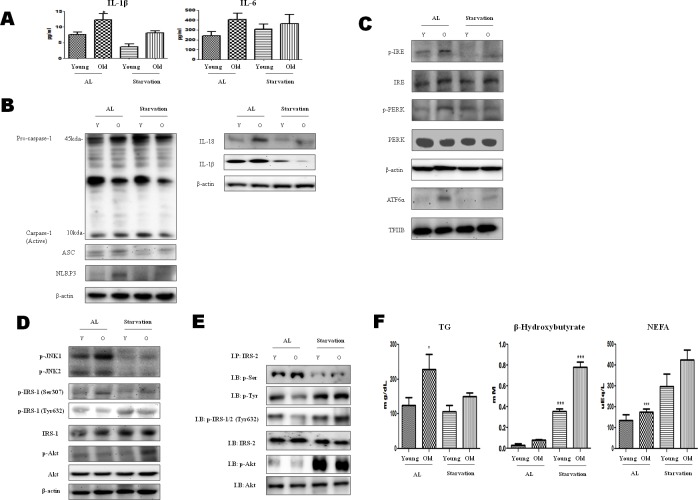
Modulation of ER-stress-induced inflammasome formation in acute starvation rat liver **A.** IL-1β and IL-6 levels were determined in the serum. Results of one-factor ANOVA: ^*^p < 0.05 *vs*. *Ad libitum*. **B.** Western blot analysis was performed to determine the inflammasome levels in the cytoplasmic extracts. As shown, the protein levels of the inflammasome were decreased by acute starvation. **C.** Western blot analysis was performed to determine the protein levels of UPR markers. As shown, the levels were significantly decreased in the starvation model. One representative blot of each protein is shown from three experiments that yielded similar results. **D.** Western blot analysis was performed to detect the presence of insulin signaling in the cytoplasmic extracts. The protein levels of serine phosphorylation of IRS-1 and phosphorylation of Akt were decreased by starvation. In contrast, tyrosine phosphorylation of IRS-1 was increased. **E.** IRS-2 interaction of Akt and its modulation by starvation. cytosol extracts were prepared from young and old rat livers. Immunoprecipitated IRS-2 was determined to be physically associated with p-seine, p-tyrosine, p-IRS-1/2 (t632), p-Akt, and Akt by Western blotting. **F.** Constituents were determined in the serum. Results of one-factor ANOVA: ^*^p < 0.05 *vs*. *Ad libitum*.

ER stress activates the NLRP3 inflammasome that is associated with the maturation of proinflammatory cytokines. Therefore, we measured the proinflammatory cytokine (IL-6 and IL-1β) levels in 3-day-starved aged rats. These cytokines have a central role in the pathology of chronic inflammatory diseases. The results showed that IL-1β increased with age, and this increase was significantly suppressed by the starvation (Figure 1A). These data suggest the possibility of a relation between acute starvation and the inflammasome.

The inflammasome complex is composed of NLRP3, caspase-1, and apoptosis-associated speck-like protein containing a caspase activation and recruitment domain (ASC). The oligomerization of NLRP3 and the recruitment of ASC and procaspase-1 trigger the autoactivation of caspase-1, which in turn cleaves other cytosolic targets, including the proinflammatory cytokines IL-1β and IL-18 [15]. To investigate inflammasome formation induced by acute starvation, the levels of caspase-1, ASC, NLRP3, IL-1β, and IL-18 were examined. As shown in Figure 1B, the levels of each of these biomolecules were increased in old rats, whereas their formation was decreased by starvation.

To characterize the molecular mechanisms underlying the effects of acute starvation, western blot analysis was performed. Markers of ER stress (*viz*., phosphorylated protein kinase (PKR)-like endoplasmic reticulum kinase (p-PERK), phosphorylated endoplasmic reticulum to nucleus signaling 1 (p-IRE1), and ATF6α) were increased in the old rats, but activated forms of ER stress were decreased under starvation conditions. These results indicate that acute starvation ameliorated the ER stress (Figure 1C).

Insulin receptor substrate-1 (IRS-1) and Insulin receptor substrate-2 (IRS-2) are substrates for the insulin receptor tyrosine kinases. ER stress leads to the suppression of insulin receptor signaling through the hyperactivation of c-Jun N-terminal kinase (JNK) and the subsequent serine phosphorylation of IRS-1 [16]. IRS-1 and IRS-2 and PI3K/Akt are important markers of insulin receptor signaling. Thus, enhancing the phosphorylation of Akt could alleviate the insulin resistance. These data indicate that acute starvation improves insulin resistance through the suppression of ER stress (Figure 1D). In our current immunoprecipitation study, the interaction between tyrosine phosphorylation of IRS-2 and phosphorylation of Akt decreased during aging, but was increased by starvation (Figure 1E). These results suggest that the aging process may induce the serine phosphorylation of IRS-1 and IRS-2, and be reversed by starvation.

On the other hand, to examine the metabolic changes caused by acute starvation in aged rats, the β-hydroxybutyrate, triglyceride (TG), and non-esterified fatty acid levels were measured. The TG level was increased during aging, but was decreased by starvation. In contrast, the ketone body increased in the starvation group (Figure 1F). To further examine the β-hydroxybutyrate effects on ER stress and inflammasome formation, the next experiments were conducted.

### β-Hydroxybutyrate had positive potential to ameliorate inflammation

**Figure 2:**
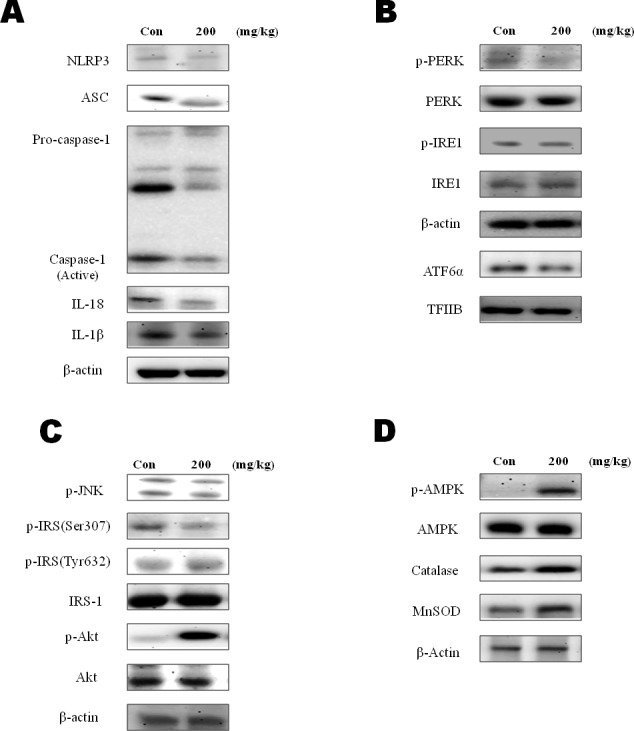
Modulation of β-hydroxybutyrate on ER-stress-induced inflammasome formation and the AMPK pathway **A.** Western blot analysis was performed to detect the presence of caspase-1, ASC, NLRP3, β-actin, IL-18, and IL-1β in liver homogenates from β-hydroxybutyrate-treated rats (200 mg·kg^−1^·day^−1^ for 5 d). **B.** To determine ER stress marker changes by β-hydroxybutyrate treatment, the expressions of p-PERK, p-IRE1, and ATF6α were examined. **C.** Western blot analysis was performed to detect the presence of phosphorylated IRS-1 at Ser 307 and Tyr 632, phosphorylated Akt, and phosphorylated JNK in liver homogenates. **D.** Western blot analysis was performed to detect the presence of p-AMPK, catalase, and SOD-2 (MnSOD) in liver homogenates.

To examine the effect of β-hydroxybutyrate on the inflammasome, protein levels of the inflammasome components were determined using western blot analysis. The levels of caspase-1, IL-18, and IL-1β were decreased in the β-hydroxybutyrate-injected groups. Interestingly, the ASC and NLRP3 components of the inflammasome were decreased by β-hydroxybutyrate treatment (Figure 2A).

As shown in Figure 2B, β-hydroxybutyrate inhibited the ER stress markers. The ER stress mediated enhancements of ATF6α, p-PERK, and p-IRE1 were also decreased in the β-hydroxybutyrate-treated group. Moreover, β-hydroxybutyrate suppressed the ER stress associated with insulin signaling. β-Hydroxybutyrate inhibited p-JNK, which induced the Ser 307 phosphorylation of IRS-1. β-Hydroxybutyrate also increased Akt phosphorylation in the insulin signaling pathway. These results suggest that β-hydroxybutyrate has the potential to improve insulin signaling (Figure 2C).

In advance, β-hydroxybutyrate suppressed the inflammasome by reducing ER stress. AMPK is widely recognized to be a key regulator of fatty acid and glucose homeostasis. It is also reported to possess protective effects against ER stress [17]. We next investigated whether β-hydroxybutyrate activates AMPK. The result showed that β-hydroxybutyrate significantly increased the phosphorylation of AMPK (Figure 2D). As FOXO3 transcription factors are known to be increased by AMPK, our findings indicate that FOXO3 activation is involved in oxidative stress. MnSOD and catalase, two major antioxidant enzymes that play a role in the protection against oxidative stress, were also found to be increased by the β-hydroxybutyrate treatment.

### β-Hydroxybutyrate suppressed the inflammasome and ER stress *via* AMPK activation

**Figure 3:**
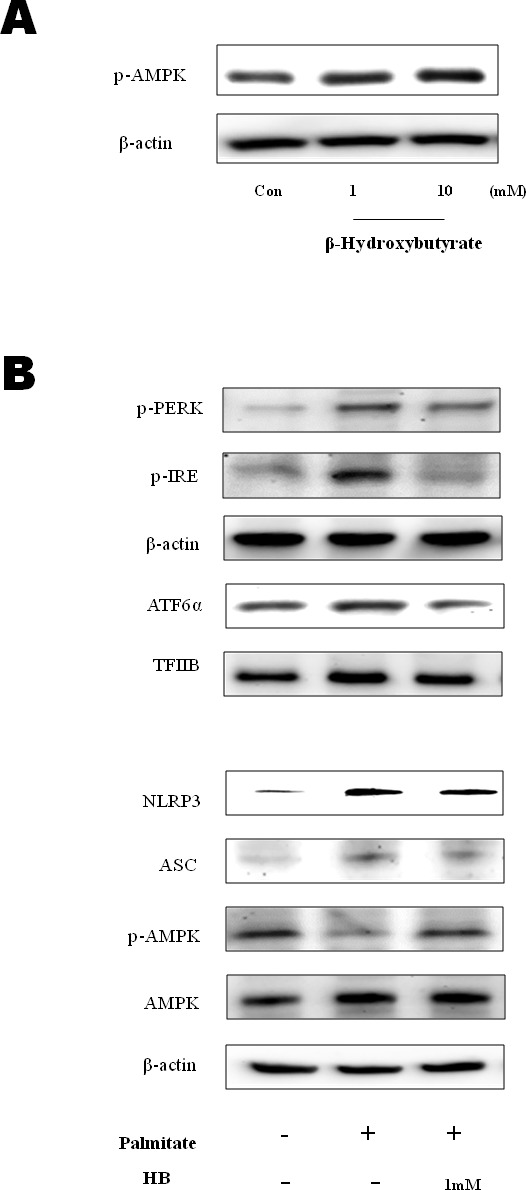
Effect of β-hydroxybutyrate on AMPK activation and ER-stress-induced inflammasome formation in HepG2 cells **A.** HepG2 cells were treated with β-hydroxybutyrate (1 and 10 mM). Cells were lysed after 2 h of treatment, and western blot analysis was performed to determine the change of phosphorylation of AMPK in the cytosolic fraction. **B.** HepG2 cells were treated with 1 mM β-hydroxybutyrate and incubated. After 2 h, the cells were treated with 250 μM palmitate. Levels of p-PERK, p-IRE1, ATF6α, NLRP3, ASC, p-AMPK, and AMPK were then measured by western blot analysis using their specific antibodies. β-Actin and TFIIB blot analyses are shown, to clarify the same amount of proteins loaded in the cytosolic and nuclear fractions, respectively.

Edwards et al., [18] recently reported that β-hydroxybutyrate suppressed inflammation through AMPK activation. To determine whether β-hydroxybutyrate inhibits inflammation by AMPK activation, cell experiments were performed. As shown in Figure 3A, AMPK phosphorylation was increased in the β-hydroxybutyrate-treated HepG2 cells. Palmitate, which is one of the FFAs, alters the ER environment, causing misfolded proteins to be produced. It is possible that the accumulation of misfolded proteins in the ER in turn activates the UPR [19]. To investigate whether ER stress is reduced by β-hydroxybutyrate, the protein levels of ER stress markers were determined. p-PERK, p-IRE1, and ATF6α were increased in the palmitate-treated HepG2 cells, whereas these marker levels were reduced by β-hydroxybutyrate treatment. In addition, β-hydroxybutyrate decreased the levels of NLRP3 and ASC. On the other hand, the phosphorylation of AMPK was increased by β-hydroxybutyrate, which was further studied as described below (Figure 3B).

### β-Hydroxybutyrate inhibited the ER-stress-induced inflammasome *via* the AMPK pathway

**Figure 4:**
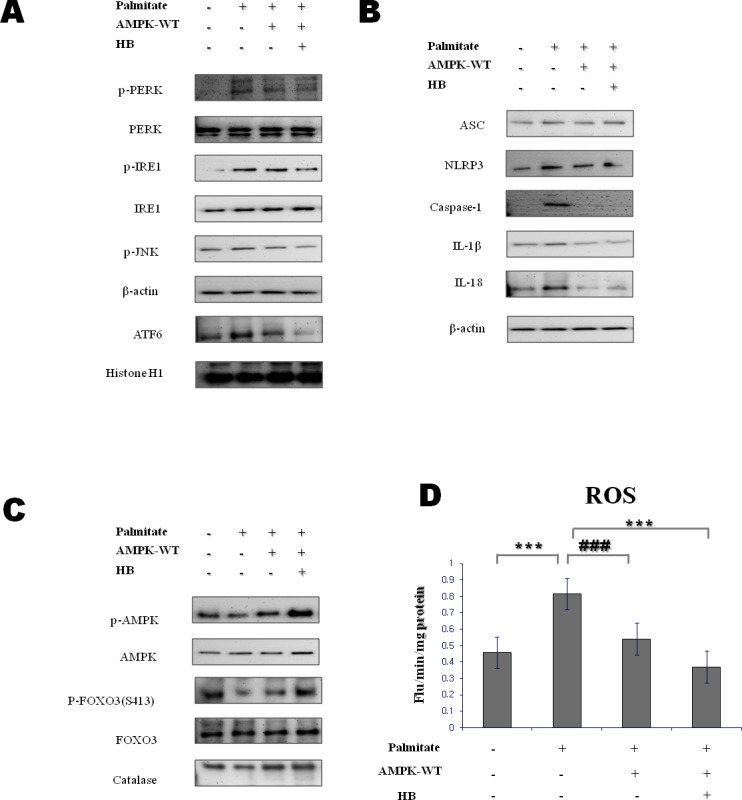
Effect of β-hydroxybutyrate on ER-stress-induced inflammasome formation *via* the AMPK pathway *in vitro* **A.** HepG2 cells were transfected with lentiviral particle wild-type AMPK. After 12 h, cells were treated with 1 mM β-hydroxybutyrate, followed by 250 μM palmitate for 1 h. Western blot analysis was performed to determine the levels of p-PERK, p-IRE1, p-JNK, and ATF6α. β-Actin and histone H1 blots are shown, to clarify the same amount of protein loaded in the cytosolic and nuclear fractions, respectively. **B.**, **C.** HepG2 cells were transfected with lentiviral particle wild-type AMPK. After 12 h, cells were treated with 1 mM β-hydroxybutyrate, followed by 250 μM palmitate for 1 h. **D.** Reactive oxygen species generation was measured by dichlorofluorescein formation with the fluorescent probe 2′,7′-dichlorofluorescein diacetate. Results of one-factor ANOVA: ^***^p < 0.001 *vs*. control; ^###^*p* < 0.001 *vs*. palmitate. HB, β-hydroxybutyrate.

AMPK has been shown to play a critical role in controlling the systemic energy balance and metabolism [20]. Furthermore, AMPK may be a physiological regulator that maintains ER homeostasis [21]. To confirm the direct involvement of AMPK activation in ER stress amelioration by β-hydroxybutyrate, we examined the effect of β-hydroxybutyrate on ER stress. For these experiments, transient transfection with expression vectors for wild-type AMPK was performed. AMPK caused the reduction of ER stress markers, which were also further reduced by treatment with β-hydroxybutyrate (Figure 4A).

AMPK activation leads to the inhibition of ROS generation and attenuates the activation of the inflammasome and the release of IL-1β [22]. To further clarify the role of AMPK activation in the inflammasome suppression by β-hydroxybutyrate, the AMPK-WT lentiviral particle was used. As shown in Figure 4B, β-hydroxybutyrate suppressed the inflammasome *via* AMPK activation. Likewise, IL-1β and IL-18 were decreased by β-hydroxybutyrate.

It is reported that age-related inflammasome can be suppressed by AMPK-mediated FOXO3 phosphorylation [23]. Therefore, we examined relationship between inflammasome and AMPK-induced FOXO3 activation. β-Hydroxybutyrate increased the phosphorylation of AMPK, and the levels of phosphorylated FOXO3 (Ser 413) were increased by AMPK in cells. Furthermore, β-hydroxybutyrate increased the antioxidant protein catalase. It is therefore considered that β-hydroxybutyrate can reduce oxidative stress by increasing catalase *via* the AMPK-FOXO3 pathway (Figure 4C).

ROS generation triggers inflammasome activation. To investigate the β-Hydroxybutyrate effect on oxidative stress *via* AMPK activation, ROS were measured using DCFDA. β-Hydroxybutyrate reduced ROS levels through AMPK activation (Figure 4C). These results suggest that β-hydroxybutyrate suppresses the palmitate-treated inflammasome *via* AMPK activation.

## DISCUSSION

β-Hydroxybutyrate, which is increased after fasting and CR, has beneficial effects [6]. Ketogenesis begins within 24 h of fasting, through gluconeogenesis [24]. Ketogenic diets, which are used in certain weight reduction programs and have been used to treat patients with refractory epilepsy, contain at least 50% of calories as fat [3]. Mild ketosis is used as a treatment for Alzheimer's disease. Recent study showed that CR increased the β-hydroxybutyrate concentration as compared with feeding *ad libitum* [6].

Diabetic ketoacidosis and the hyperglycemic hyperosmolar non-ketotic state are two serious metabolic complications of diabetes [25]. Likewise, ketone bodies such as β-hydroxybutyrate can be toxic when present at very high concentrations in individuals with diseases such as type I diabetes. On the other hand, β-hydroxybutyrate helps to protect cells from oxidative stress at lower concentrations [6]. It also appears to have broadly neuroprotective effects in these and other neurodegenerative disease models [26]. However, β-hydroxybutyrate inhibited NLRP3 inflammasome in macrophages such as immune response [27].

Proteins that are unable to fold correctly cause ER stress and activate the UPR [28]. Additionally, ER stress activates the NLRP3 inflammasome [29], a molecular platform to trigger the innate immune defense through the maturation of proinflammatory cytokines. Upon detection of a cellular stress, such as K^+^ efflux, lysosomal rupture, and ROS, NLRP3 recruits ASC and procaspase-1, which results in caspase-1 activation and the processing of cytoplasmic targets, including IL-1β and IL-18 [30]. Inflammasome activation has been linked to human diseases such as type 2 diabetes [31] and obesity [32]. The present study focused on the inhibitory effects of β-hydroxybutyrate on ER-stress-induced inflammasome formation.

ER stress has also emerged as an important player in hepatic insulin resistance, leading to the development of hyperglycemia and hyperlipidemia. ER stress can inhibit insulin signaling. IRE1 activation (an ER stress marker) leads to the phosphorylation of JNK, which impairs insulin signaling by phosphorylating IRS-1 Ser 307 [33]. In this study, ER stress and p-JNK were increased in aged rats, but were reduced by starvation (Figure 1). These observations suggest that acute starvation ameliorates age-related inflammation through the inhibition of ER stress and the inflammasome.

The inflammasome is associated with AMPK downregulation. In other studies, activated AMPK attenuated protein translation, leading to a reduction of the ER and an alleviation of ER stress [34]. Interestingly, mice treated with β-hydroxybutyrate showed increased expression of FOXO3, MnSOD, and catalase, which protect against oxidative stress in kidney tissues [6, 35]. FOXO has a protective role in resisting oxidative stress through the regulation of antioxidant genes (MnSOD and catalase) as well as additional cell survival pathways [36]. Nevertheless, the effects of β-hydroxybutyrate on ER stress and inflammasome formation in the liver are unclear. In the present study, the expressions of p-AMPK, MnSOD, and catalase in rats were increased by β-hydroxybutyrate treatment (Figure 2D). These data suggest that β-hydroxybutyrate activates AMPK, leading to the upregulation of MnSOD and catalase activation. β-Hydroxybutyrate also suppressed inflammasome and ER stress indicators, thus demonstrating its protective effect against the inflammasome and ER stress.

To clarify the effects of β-hydroxybutyrate on ER stress and the inflammasome *via* AMPK activation, AMPK-WT lentiviral particle treatment was examined *in vitro*. According to the experimental results, β-hydroxybutyrate reduced the inflammasome and ER stress *via* AMPK-FOXO3 signaling. The decline of inflammasome activation seems to be regulated by ROS reduction through catalase activation, which is a downstream gene of FOXO3 (Figure 4). This experimental result might contribute to improving the suppression of the inflammasome, which is one of the causes of age-related diseases.

In summary, β-hydroxybutyrate, which is generated in the process of gluconeogenesis by acute starvation, inhibited inflammasome formation through suppression of protein expression of ER stress. Additionally, β-hydroxybutyrate increased MnSOD and catalase by mediating FOXO3 through AMPK activation. Thus, these changes imply that ROS generation by β-hydroxybutyrate might prevented inflammasome activation.

In conclusion, β-hydroxybutyrate reduced oxidative stress via AMPK signaling, leading to reduction of ER stress and inflammasome. Future studies will focus on the potential applications of these findings in the prevention of ER stress and inflammasome, and associated complications.

## MATERIALS AND METHODS

### Animals

Specific pathogen-free (SPF) male Sprague Dawley (SD) rats were obtained from Samtako (Osan, Korea) and were fed a standard laboratory diet (Superfeed Co., Wonju, Kangwon, Korea) *ad libitum*. The animals at 6 and 24 months of age were used as young and old rats, respectively. There were 6 rats in each experimental group. The *ad libitum* (AL)-fed group had free access to both food intake of their AL-fed littermates and the starvation practiced for 3 d. To estimate the effects of β-hydroxybutyrate on ER stress and inflammasome formation, β-hydroxybutyrate was injected once per day for 5 d at a dose of 200 mg·kg^−1^·d^−1^ to 8-week-old SD rats. This concentration was selected based on a previous study of the regulation of sympathetic nervous system activity by ketone bodies [37]. All animal studies were designed by the Aging Tissue Bank and approved by the Institutional Animal Care Committee of Pusan National University. We followed the guidelines for animal experiments issued by Pusan National University (Approval Number PNU-2012-0088).

### Cell culture system

The human hepatoma cell line (HepG2) was obtained from the American Type Culture Collection (ATCC, Manassas, VA, USA). The cells were grown in Dulbecco's modified Eagle's medium (DMEM; Nissui, Tokyo, Japan) containing 2 mM l-glutamine, 100 mg/mL streptomycin, 2.5 mg/L amphotericin B, and 10% heat-inactivated fetal bovine serum (FBS). The cells were maintained at 37°C in a humidified atmosphere containing 5% CO_2_. Cells that had been in culture for more than 3 months were not used for the experiments; instead, new cells were obtained from frozen stock. Cells at the exponential phase were used for all experiments.

### Free fatty acid preparation

A stock solution of 100 mM palmitate (Sigma-Aldrich, USA) was prepared in 0.1 M NaOH at 70°C and filtered. A 5% (wt/vol) FFA-free BSA (Sigma-Aldrich) solution was prepared in double-distilled H_2_O and filtered. A 5 mM FFA/5% BSA solution was prepared by combining the appropriate amount of FFA to 5% BSA in a 60°C water bath. This solution was then cooled to room temperature and diluted 1:20 in DMEM without FBS to a final concentration of 1 mM FFA/1% BSA [19].

### Biochemical analysis

Blood samples were collected after the animals in each group had been sacrificed. Commercial kits were used to measure the concentration of triglyceride (Sinyang Co, Korea) and FFAs in the serum. The serum FFA level was determined using the assay kit SICDIA NEFAZYME (Sinyang Co, Korea). IL-1β and IL-6 were measured using the Luminex multiplex cytokine system. β-Hydroxybutyrate concentrations were determined using a β-hydroxybutyrate detection kit (Stanbio Laboratory, USA).

### Western blot analysis

Homogenized liver tissues and lysed cell samples were boiled for 5 min with a gel-loading buffer (pH 6.8, 125 mM Tris-HCl, 4% sodium dodecyl sulfate (SDS), 10% 2-mercaptoethanol, and 0.2% bromophenol blue) in a ratio of 1:1. Equal amounts of protein were separated by sodium dodecyl sulfate-polyacrylamide gel electrophoresis, using 6-17% gels. The gels were subsequently transferred onto an Immobilon-P transfer membrane (Millipore Corp, Bedford, MA, USA). The membrane was immediately placed in a blocking solution (5% non-fat dry milk in TBS-Tween (TBS-T) buffer containing 10 mM Tris, 100 mM NaCl, and 0.1% Tween 20, pH 7.5) at room temperature for 1 h. The membrane was washed in TBS-T buffer for 30 min and incubated with the primary antibody at room temperature for 2 h. After a 30-min wash in TBS-T buffer, the membrane was incubated with a secondary antibody at room temperature for 1 h. After a 40-min wash in TBS-T buffer, the antibody labeling on the membrane was detected using the enhanced chemiluminescence method according to the manufacturer's instructions, and exposed on radiographic film. Prestained blue protein markers were used for the molecular weight determination.

### Measurement of intracellular reactive oxygen species levels

HepG2 cells in a 96-well plate were preincubated for 24 h. After 1 d, the medium was replaced with fresh serum-free medium. Cells were then either treated with β-hydroxybutyrate (1 or 5 μM) or not treated, and incubated for 1 h. After treating with 100 mM glucose for 1 h, the medium was replaced with fresh serum-free medium and 125 μM 2′,7′-dichlorofluorescein diacetate (DCFDA) was added. The fluorescence intensity of the dichlorofluorescein was measured for 35 min using the Fluorescence Plate Reader with excitation and emission wavelengths of 485 and 535 nm, respectively.

### Transient transfection

The AMPK-WT pcDNA3 expression vectors for wild-type and mutant rat AMPKα2 were a generous gift from Prof. Myeong Ho Jung (Department of Korean Medicine, Pusan National University, Busan, Korea). HepG2 cells were grown in 6-well culture plates to approximately 60-70% confluence. The cells were then transfected with pcDNA-AMPK-WT.

### Reactive species (RS) measurements

To quantify intracellular RS generation, HepG2 cells were seeded in a 96-well plate. One day later, the medium was changed to fresh serum-free medium. HepG2 cells were transfected with lentiviral particle wild-type AMPK. After 12 h, cells were treated with 1 mM β-hydroxybutyrate, followed by 250 μM palmitate for 1 h, and medium was replaced with fresh serum free medium containing DCFDA (f.c. 2.5 μM). Measurement of the fluorescence intensity of DCF was performed every 5 min for 30 min using a microplate fluorescence reader TECAN (Salzburg, Austria) using excitation and emission wavelengths of 485 and 535 nm, respectively.

### Immunoprecipitation assay

Cytosol extracts were subjected to immunoprecipitation (IP) in a buffer containing 40 mM Tris-HCl (pH 7.6), 120 mM NaCl, 20 mM β-glycerophosphate, 20 mM NaF, 2 mM sodium orthovanadate, 5 mM EDTA, 1 mM PMSF, 0.1% NP40 with leupeptin (2 ug/ml), aprotinin (1 ug/ml), and pepstatin A (1 ug/ml). One thousand micrograms of cytosol extracts were incubated with 50% slurry of protein A agarose for 30 min at 4°C for preclearing. After incubation, cytosol extracts were centrifuged at 12,000 g at 4°C for 5 min. The cytosol extracts were then incubated overnight with the respective antibody at 4°C followed by incubation overnight at 4°C with 50% slurry of protein A agarose. After washing of the immunoprecipitates three times with IP buffer, the immunoprecipitated proteins were analyzed by SDS-PAGE followed by Western blotting as described previously.

### Statistical analysis

One-way analysis of variance (ANOVA) was used to analyze differences among three or more groups. Differences in the means of individual groups were assessed by Bonferroni's *post hoc* test. *p*-values < 0.05 were considered statistically significant. Analysis was performed using GraphPad Prism 5 software (La Jolla, CA, USA).

## Abbreviations

ER: Endoplasmic reticulum
NLRP3: inflammasome NOD-like receptor protein 3 inflammasome
AMPK: AMP-activated protein kinase
FOXO3: Forkhead O bax transcription factor 3
HB: β-hydroxybutyrate
